# Computational Design of Anticorrosion Properties of Novel, Low-Molecular Weight Schiff Bases

**DOI:** 10.3390/ma15196725

**Published:** 2022-09-27

**Authors:** Szymon Malinowski

**Affiliations:** Department of Construction Materials Engineering and Geoengineering, Faculty of Civil Engineering and Architecture, Lublin University of Technology, Nadbystrzycka 40, 20-618 Lublin, Poland; s.malinowski@pollub.pl; Tel.: +81-538-4451

**Keywords:** corrosion, Density Funtional Theory (DFT), Schiff bases, electron transfer, interaction energy, corrosion inhibitor

## Abstract

Due to the many economic consequences and technological problems caused by the corrosion process, its inhibition is one of the most important aspects of ongoing research. Computer methods, i.e., density functional theory (DFT) methods, are of great importance to the large-scale research being conducted which allows the evaluation of the corrosion inhibition performance without conducting time-consuming, long-term and expensive experimental measurements. In this study, new corrosion inhibitors were designed in three corrosion environments on the basis of their HOMO and LUMO orbital energies—the energy difference between them and their dipole moment. In addition, their interactions with the Fe and Cu surface were modelled on the basis of the number of electrons transferred during the formation of the protective adsorption layer (ΔN) and the initial energy between inhibitor molecule and protected metal surface (Δψ). The obtained results indicate that, among the aliphatic investigated Schiff bases, the N-methylpropan-1-imine (N-MP(1)I) molecule would theoretically have the highest corrosion inhibition efficiency mainly due to its high E_HOMO_ value, relatively low E_LUMO_ value, high chemical reactivity and high polarity.

## 1. Introduction

Corrosion is a process leading to the degradation of materials, especially metallic materials [1,2,3]. This process occurs mainly through its interaction with the surrounding natural environment. According to the definition introduced by IUPAC (International Union of Pure and Applied Chemistry), corrosion is a process occurring as a result of chemical/electrochemical reaction at the corroding material–environment phase boundary [4]. The corrosion process is impacted by a number of factors that include the nature of the corroding metal and the type of the corrosive medium. The direct consequence of corrosion is loss of useful properties, with consequences especially important for the construction, chemical, automotive or metallurgical [1,5] industries. They are mainly related to the loss of material and the high financial expenses required for their corrosion protection [3,5,6]. The latest data presented by the National Association of Corrosion Engineers (NACE) indicate that corrosion-induced losses reach about $2.5 trillion [7].

Many different methods are used to protect metals from corrosion, which include isolation of the corroding metal from corrosive agents (protective coatings, film-forming chemicals), compensation of charge loss due to corrosion (cathodic protection), electroplating [8,9] and application of corrosion inhibitors. The corrosion inhibitors are organic and inorganic compounds that, in low concentrations, reduce the chemical and/or electrochemical reactions of the metal with the aggressive environment [5,10,11,12]. Limitation of the corrosion process happens due to their chemical (chemisorption) and/or physical adsorption. Consequently, a protective layer that separates the corroding metal from the harmful corrosive factors existing in the surrounding environment is formed [12]. Moreover, limitation of the corrosion process can result from increase or decrease of the anodic and/or cathodic electrochemical corrosion reaction as well as minimization of corrosion reaction reactants diffusion rate [13]. A potential corrosion inhibitor for use in real corrosion systems must be readily available, environmentally friendly, renewable, and its use must be economically justified [14]. The performance of corrosion inhibitors depends mainly on their chemical and electron structure including electron density, number of free electron pairs and number of π electrons [3,15].

The corrosion inhibition efficiency of newly-synthesized inhibitors is currently being studied with experimental approaches using mainly gravimetric or electrochemical methods [5,16,17,18,19]. However, these methods are relatively time-consuming, as they require long conditioning of the metal in a corrosive environment. Therefore, in recent years, computer-based methods have become increasingly popular in the design of modern corrosion inhibitors [20,21,22,23,24]. These methods provide fast, accurate and precise analysis of the organic compounds’ inhibition ability based on their electronic structure [25]. Among the computer methods widely used in the design of modern corrosion inhibitors, DFT (Density Functional Theory) methods are very important and commonly employed [26,27,28]. As demonstrated in numerous studies by classical experimental and DFT methods presented in the literature, there are strong correlations between corrosion inhibition performance of organic compounds and its electron parameters, i.e., HOMO orbital energy or LUMO orbital energy [24,29,30].

**Scheme 1:**
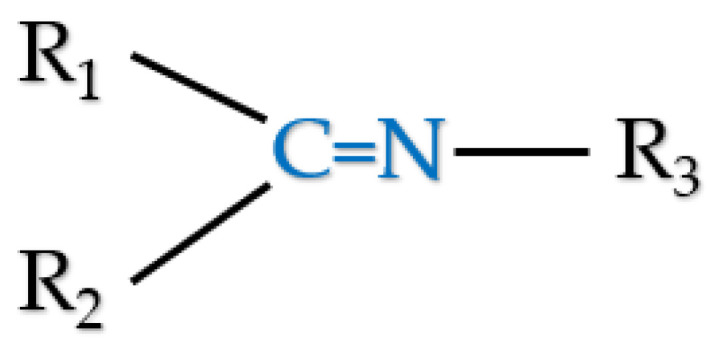
General chemical structure of Schiff bases.

In the corrosion protection of metals, compounds with nitrogen atoms are very important because of the free electron pair. Therefore, many experimental and theoretical studies were used to investigate the corrosion inhibition efficiency of chemical compounds from the Schiff’s base group. As is shown in Scheme 1, Schiff bases are organic compounds that have a characteristic chemical structure [31] based on a C=N bond with three independent hydrocarbon chains (R_1_, R_2_ and R_3_), where R_3_ cannot be an H atom [32]. In general, they are considered a subclass of imines in which the H atom (R_3_) has been replaced by any aliphatic or aromatic substituent. This characteristic C=N bond is the main factor responsible for the anticorrosive properties of Schiff bases due to formation of strong interactions, with ions present in the crystal lattice of the metal [32]. This is due to two structural factors: (1) the free electron pair on the N atom and (2) the unsaturated bond C=N [33]. Following the current trends of research work on the design of modern corrosion inhibitors, the aim of this work is detailed electron properties analysis of 12 aliphatic Schiff bases in order to select the compound with the theoretically highest anti-corrosion properties. For this purpose, the analysis was carried out in 4 corrosion media, i.e., gas phase, aqueous phase, HCl solution with a concentration of 1 mol/dm^3^ and 2 mol/dm^3^, considering the following structural aspects:

1. the length of the aliphatic chain attached to the N atom (R_3_);
2. the length of the aliphatic chain attached to the C atom (R_1_) when the -CH_3_ substituent is attached to the N atom;
3. the length of the aliphatic chain attached to the C atom (R_1_) when the substituent -C_2_H_5_ is attached to the N atom;
4. the length of the aliphatic chain attached to the C atom (R_1_) when the substituent -C_3_H_7_ is attached to the N atom.

## 2. Theory and Calculation Method

The quantum-chemical calculations presented in the paper were carried out using the parallel quantum solutions (PQS) suite of ab initio programs and the PQSmol Graphical user interface package at B3LYP theory level at 6-311g-dp basis set. This work involves the analysis of the electron structures of 12 aliphatic Schiff bases with different chemical structures. The structural formulas, names and abbreviations of those used in the work are summarized in Table 1. The effect of the chemical structure of the studied corrosion inhibitors on their electronic properties and, consequently, on the anticorrosion properties was carried out for the gas phase, aqueous phase as well as for an HCl solution with a concentration of 1 mol/dm^3^ and 2 mol/dm^3^. The electronic properties of the studied Schiff bases in H_2_O and HCl phase were determined based on quantum-chemical calculations carried out using the COSMO (Conductor-like Screening Model) solvent model. This was performed in order to obtain the realistic parameters of the corrosion system as accurately as possible during the optimization of the chemical structures of the studied Schiff bases in an HCl environment with a concentration of 1 mol/dm^3^ and 2 mol/dm^3^ applying the electrical permittivity value of 51.4 and 30.2, respectively [34].

**Scheme 2:**
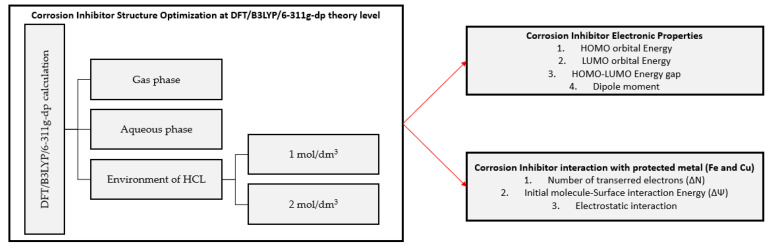
Overall scheme of performed DFT calculations.

**Figure 1:**
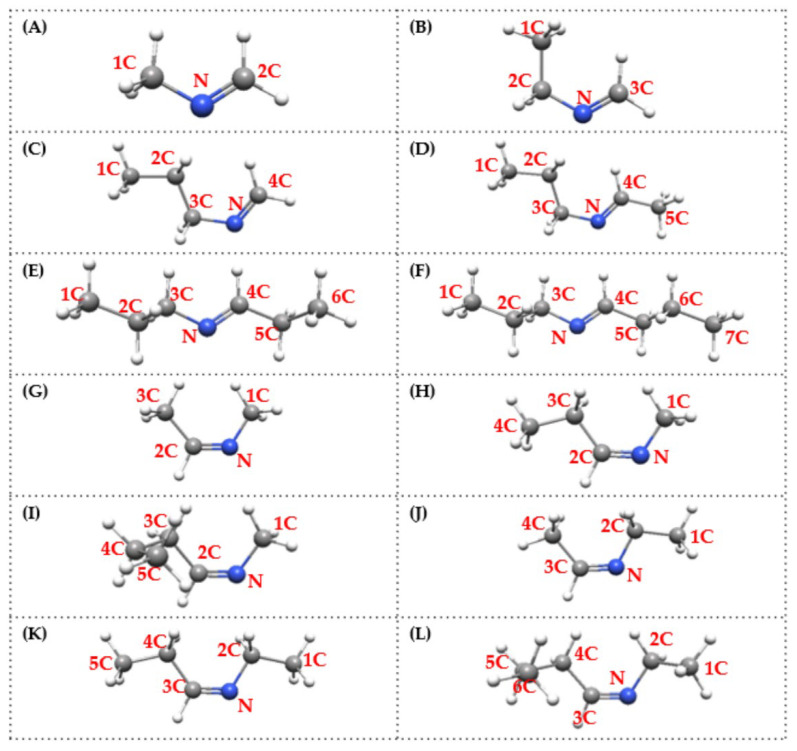
Optimized structure of studied aliphatic Schiff bases, (**A**) N-MMI, (**B**) N-EMI, (**C**) N-PMI, (**D**) N-PEI, (**E**) N-PP(1)I, (**F**) N-PB(1)I, (**G**) N-MEI, (**H**) N-MP(1)I, (**I**) N-MP(1)I, (**J**) (1Z)N-EEI, (**K**) (1Z)N-EP(1)I, (**L**) (1Z)N-EB(1)I.

The validity of the optimized chemical structures of the studied aliphatic Schiff bases, shown in Figure 1, was confirmed on the basis of the IR spectra generated by the lack of imaginary frequencies with negative wave number values. As is seen in Scheme 2, their anticorrosion properties were analyzed based on the energy of the HOMO (The Highest Occupied Molecular Orbital) orbital, the energy of the LUMO (The Lowest Unoccupied Molecular Orbital) orbital, the energy difference between them (ΔE) determined using Equation (1) and the dipole moment (μ). To study interactions of investigated aliphatic Schiff bases with a metal surface, the number of transferred electrons (ΔN) and initial metal–molecule interaction energy (ΔΨ) were determined using Equations (2) and (3). Meanwhile, the electronegativity (χ) and global hardness (η) values necessary to determine ΔN and ΔΨ were calculated using the Formulas (4) and (5), respectively. According to data from the literature, for Fe and Cu surfaces, χ values were respectively 7.94 eV [20] and 4.48 eV [35]. In the last step, an analysis of electrostatic interactions at the interface between the investigated inhibitor molecule and the protected metal surface was carried out on the basis of partial charges determined by Mulliken population analysis.

ΔE = E_LUMO_ − E_HOMO_ (1)

(2) $$\Delta N=\frac{\chi_{\mathrm{Fe}/\mathrm{Cu}}-\chi_{\mathrm{inh}}}{2\eta_{\mathrm{inh}}}$$

(3) $$\Delta \Psi =\frac{{\left(\chi_{\mathrm{Fe}/\mathrm{Cu}}-\chi_{\mathrm{inh}}\right)}^{2}}{4\eta_{\mathrm{inh}}}\;$$

(4) $$\chi =\frac{\left(-E_{\mathrm{HOMO}}-E_{\mathrm{LUMO}}\right)}{2}$$

(5) $$\eta =\frac{\left(-E_{\mathrm{HOMO}}+E_{\mathrm{LUMO}}\right)}{2}$$

## 3. Results and Discussion

### 3.1. Anticorrosive Properties of New Aliphatic Schiff Bases

#### 3.1.1. Gas Phase

**Figure 2:**
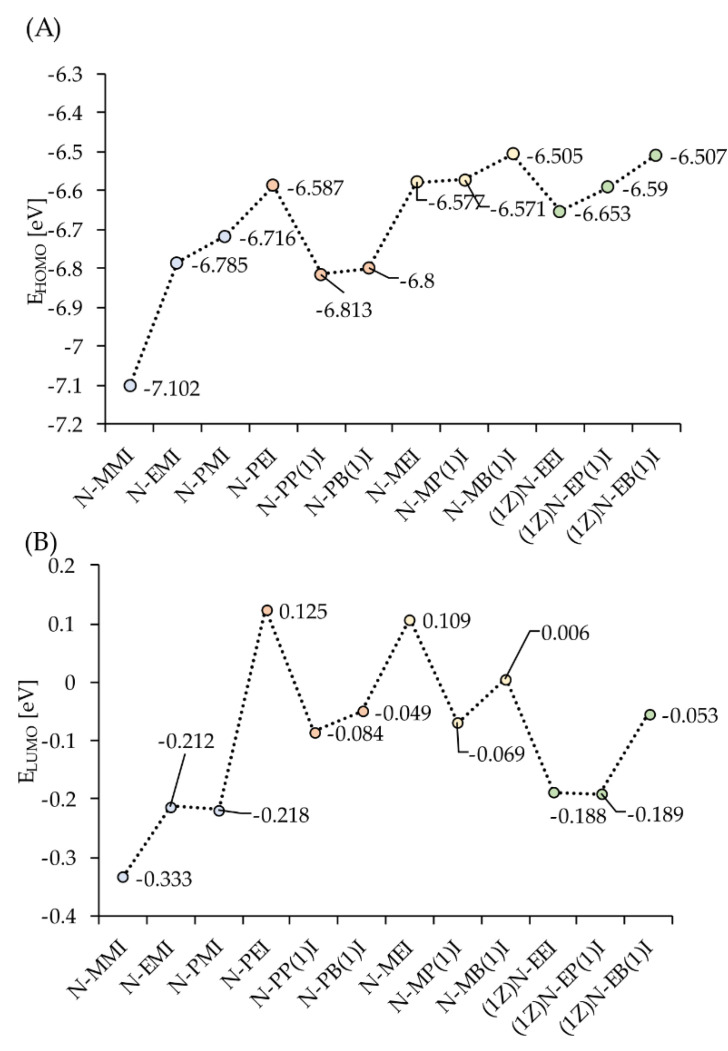
E_HOMO_ (**A**) and E_LUMO_ (**B**) of the investigated Schiff bases determined in the gas phase.

To characterize the dependence of the anticorrosion properties on the chemical structure of the investigated aliphatic Schiff bases, quantum chemical calculations at B3LYP/6-311g-dp theory level were carried out in the gas phase. First of all, their electron-donating properties were analyzed based on the value of their E_HOMO_. A higher E_HOMO_ value corresponds directly with the greater ability of the inhibitor molecule to donate electrons to the protected metal. Generally, the E_HOMO_ values of the studied aliphatic Schiff bases range from −7.102 eV to −6.377 eV. As is shown in Figure 2A, an increase in the number of carbon atoms in the substituent attached to the N atom (R_3_-Scheme 1) resulted in an increase in the E_HOMO_ values of the studied aliphatic Schiff bases. For the N-MMI, N-EMI and N-PMI molecule, E_HOMO_ values of −7.102 eV, −6.785 eV and −6.716 eV were obtained, respectively. The observed increase of more than 5% in E_HOMO_ values clearly indicates that the elongation of the aliphatic substituent R3 by the simultaneous presence of two H atoms (R_1_ and R_2_) results in an enhancement of the electron-donating properties and consequently a theoretical increase in the corrosion inhibition efficiency. Similar trends were observed for the investigated Schiff bases, with an R_3_ substituent composed of one (-CH_3_) and two (-C_2_H_5_) carbon atoms. However, the increase in E_HOMO_ values observed for these molecules was considerably lower and was approximately 1% and 2%, respectively. Totally opposite relationships were observed for elongation of the aliphatic substituent attached to the C atom (R_1_-Scheme 1) in inhibitors with the -C_3_H_7_ substituent attached to the N atom (R_3_-Scheme 1). For these compounds, a reduction in E_HOMO_ was observed from −6587 eV for the N-PEI molecule to −6800 eV N-PN(1)I. This means that aliphatic Schiff bases having a longer hydrocarbon chain R1 will have worse anticorrosion properties.

The value of the LUMO orbital energy (E_LUMO_) has also an important role in evaluating the anticorrosion properties of organic compounds as it describes the ability to accept electrons from the protected metal. Because the corrosion inhibitor protection mechanism involves the formation of a protective adsorptive layer with electron transfer at the phase boundary, molecules with a greater capacity to accept electrons will more strongly inhibit the corrosion process. The relationship between the E_LUMO_ of an organic compound and its electron-accepting capacity is inversely proportional, therefore the organic compounds with lower E_LUMO_ values are characterized by higher corrosion inhibition efficiency. As is shown in Figure 2B, among the investigated aliphatic Schiff bases with H atoms attached to the C atom (R_1_ and R_2_), the highest E_LUMO_ value was determined for N-EMI with the -C_2_H_5_ group attached to the N atom (R_3_). However, the difference in E_LUMO_ values determined for N-EMI and N-PMI molecules was negligible, only 0.006 eV. This indicates that these compounds can accept electrons from the surface of the protected metal with comparable efficiency, and consequently they will protect it from corrosion with comparable efficiency. For aliphatic Schiff bases with the -C_3_H_7_ substituent attached to the N atom (R_3_), the highest E_LUMO_ value was determined for the N-PEI molecule with the -CH_3_ group attached to the C atom (R_1_). Therefore, among these three potential corrosion inhibitors, this investigated compound is characterized by the strongest anticorrosive properties. As indicated by quantum chemical calculations carried out at a B3LYP/6-311g-dp theory level, for aliphatic Schiff bases with the -C_2_H_5_ substituent in R_3_ position, (1Z)N-EB(1)I with the -C_3_H_7_ group attached to the C (R_1_) atom has the highest corrosion inhibition ability. On the other hand, among corrosion inhibitors with the -CH_3_ group attached to the N atom (R_3_), the highest E_LUMO_ value, so the theoretically strongest anti-corrosion properties, was determined for the N-MEI molecule as also having the -CH_3_ group at the C atom (R_1_).

In summary, the electronic structure of 12 aliphatic Schiff bases determined based on quantum chemical calculations carried out at B3LYP/6-311g-dp theory level indicated that their ability to donate electrons (Figure 2A) is enhanced with an increase in the length of the aliphatic chain R_3,_ with simultaneous reduction of their electron-accepting ability (Figure 2B). The same relationship was observed for investigated aliphatic Schiff bases with -CH_3_ and -C_2_H_5_ substituent at the N atom (R_3_) during elongation of the R_1_ chain attached to the C atom, which increases the electrodonating properties (higher E_HOMO_ values), indicating stronger anti-corrosion properties (Figure 2A). Completely opposite conclusions were found in the analysis of the E_HOMO_ and E_LUMO_ values of Schiff bases with -C_3_H_5_ group attached to the N atom. For these compounds, an increase in the number of C atoms in the R_1_ substituent results in a decrease in their ability to donate electrons to the metal surface (a decrease in E_HOMO_) and an enhancement in ability to accept electrons from the metal surface (a decrease in E_LUMO_). As can be seen in Figure 2A,B, the analysis of HOMO and LUMO orbital energies is only insufficient to clearly and transparently describe the anticorrosive properties of aliphatic Schiff bases.

**Figure 3:**
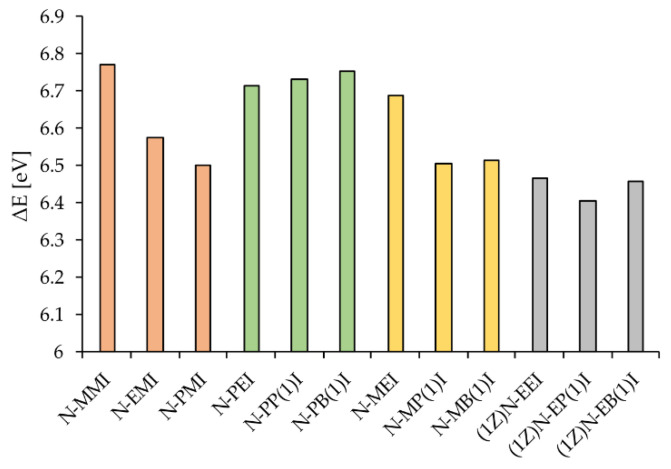
ΔE values of investigated aliphatic Schiff bases in gas phase.

The chemical reactivity and kinetic stability of organic compounds can be determined by the energy difference between the LUMO and HOMO orbitals (ΔE). Figure 3 shows that lower values of ΔE indicate a corrosion inhibitor with higher reactivity, which translates directly into better ability to form protective, anticorrosion layers on the surface of the protected metal. It means that chemical compounds with lower ΔE value will inhibit the corrosion process more effectively. The ΔE values of the studied aliphatic Schiff bases are shown in Figure 3. The obtained results indicate that for inhibitors with two H atoms at the C atom (R_1_ and R_2_), an increase in the number of C atoms building the R_3_ substituent causes a decrease in their chemical reactivity. This means that theoretically the N-PMI molecule will interact less strongly with the surface of the protected metal and adsorb on its surface. This will result in an efficiency decrease of inhibition of its corrosion process. For this group of compounds, the highest difference in ΔE values was observed, indicating that the absence of aliphatic substituents at the carbon atom (R_1_ and R_2_) makes Schiff base molecules the most sensitive to the decrease in corrosion inhibition efficiency due to the elongation of the aliphatic hydrocarbon chain attached to the N atom (R_3_). Regarding the change in chemical reactivity of the studied Schiff base molecules due to an increase in the length of the hydrocarbon chains attached to the C atom (R_1_), a decrease in the value of ΔE during the elongation of the R1 chain was observed for compounds with -CH_3_ and -C_2_H_5_ substituents at the N atom (R_3_). This decrease was less evident for the compounds (1Z)N-EEI, (1Z)N-EP(1)I and (1Z)N-EB(1)I, but simultaneously these compounds had the lowest energy difference between the LUMO and HOMO orbitals. Therefore, they inhibited the corrosion process to the lowest degree. Interestingly, only in the presence of the -C_3_H_7_ substituent attached to the N atom (R_3_) was there an enhancement of the chemical reactivity of aliphatic Schiff bases observed as a result of the elongation of the hydrocarbon chain attached to the C atom (R_1_).

As is shown in the work [32], Schiff bases synthesized using benzaldehyde and 4-4′diaminodiphenyl ether are characterized by higher E_HOMO_ values and lower E_LUMO_ values. It means that, in comparison to the aliphatic Schiff bases studied in this work, they theoretically demonstrate better anti-corrosion properties. In addition, the introduction of the -OH, -NH_2_ and -NO_2_ substituents into the structure of these compounds improved their anticorrosion properties, as indicated by an increase in the E_HOMO_ value and a decrease in the E_LUMO_ value of the obtained derivatives. Whereas, the paper [36] presents the results of thermodynamic, electrochemical and quantum chemical studies of the anticorrosive properties of Schiff bases obtained by condensing isoctinohydrazine and corresponding aldehydes in methanolic media. For the obtained compounds, namely N′-(phenylmethylene) isonicotino-hydrazide, N’-(2-hydroxybenzylidene) isonicotinohydrazide, N’-(furan-2-ylmethylene) isonicotinohydrazide and N′-(3-phenylallylidene) isonicotinohydrazide, similar E_HOMO_ and E_LUMO_ values were found. This may indicate that the aliphatic Schiff bases studied in this work may have similar corrosion inhibition efficiencies, ranging from 78.5% to 94.7% [36], but this requires further experimental verification. Schiff bases, i.e., 3-((phenylimino)methyl)quinoline-2-thiol and 3-((5-methylthiazol-2-ylimino)methyl)quinoline-2-thiol have even higher corrosion inhibition efficiency and similar E_HOMO_ and E_LUMO_ values compared to those of the compounds studied in this work. Quantum-chemical calculations performed for these compounds indicate that, compared to the aliphatic Schiff bases studied in this work, they have similar E_HOMO_ values and slightly lower E_LUMO_ values, i.e., they have comparable electron-donor properties and better electron-acceptor properties. Importantly, electrochemical and gravimetric measurements showed their very high corrosion inhibition efficiency of 96% and 99%, respectively [37]. On the other hand, the introduction of S atoms into the structure of aromatic Schiff bases results in lower E_HOMO_ and lower E_LUMO_ values, indicating at the same time that they form adsorptive protective layers on the surface of the protected metal mainly by accepting electrons from its d orbital. Gravimetric and electro-chemical studies by Mahdi et al. [38] indicated that, depending on the concentration of the inhibitor, the corrosion inhibition efficiency of this type of orbital compounds ranged from 67.1% to 92.3%. On the other hand, the phenol Schiff bases studied in the paper [39] have a higher EHOMO value and a lower ELUMO value determined in the gas phase compared to aliphatic compounds, indicating their better anticorrosion properties. This means that the aliphatic Schiff bases studied should have a lower corrosion protection efficiency than 90%. Similar corrosion inhibition efficiency and EHOMO and ELUMO values were further observed for the planar rigid pyridinecarboxaldehyde-based double-Schiff bases [40] and imidazo [1, 2-a]pyridine Schiff bases [41].

#### 3.1.2. Aqueous Phase

**Figure 4:**
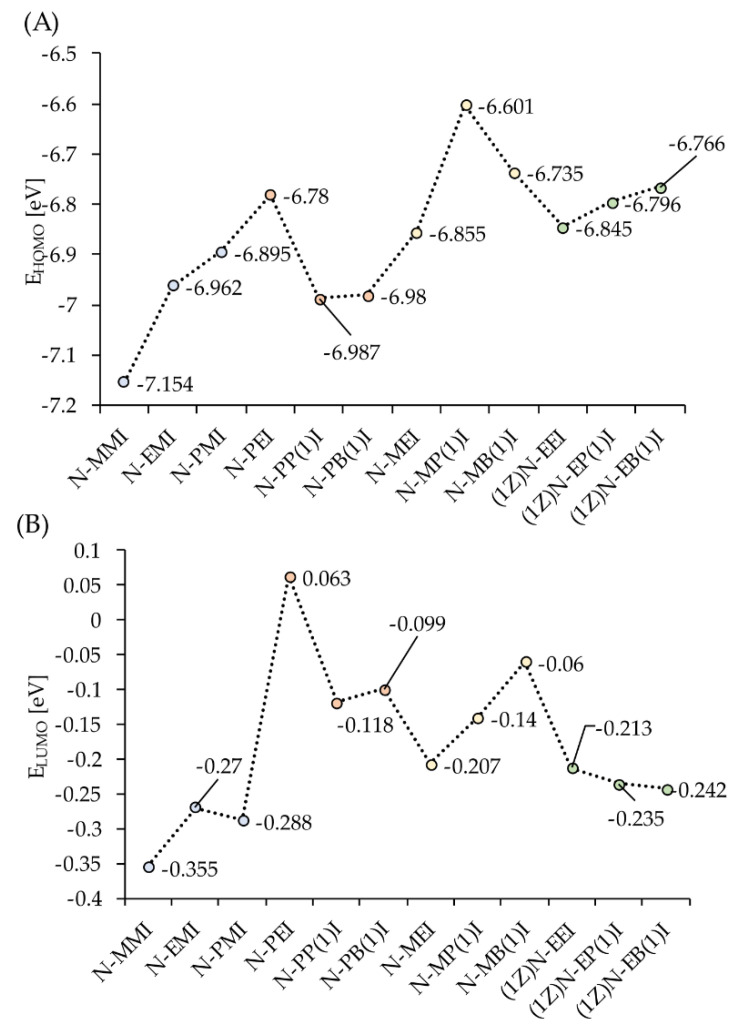
E_HOMO_ (**A**) and E_LUMO_ (**B**) of the investigated Schiff bases determined in the aqueous phase.

Because corrosion inhibitors are used in real systems in an aqueous phase, this study also involves analysis of electronic properties and resulting anti-corrosion properties of the aliphatic Schiff bases when considering the presence of H_2_O molecules. Specifically, the analysis was focused on how the electronic structure of the investigated corrosion inhibitors correlates with their sensitivity to a change in the corrosion environment. Figure 4A,B shows the energies of the HOMO and LUMO orbitals of the investigated compounds determined in an aqueous phase.

A comparison of the E_HOMO_ values of the investigated corrosion inhibitors in gaseous (Figure 2A) and aqueous (Figure 4A) phases indicates that the corrosion environment does not change the dependence of the anticorrosion properties of the aliphatic Schiff bases on their chemical structure. However, the quantum chemical calculations carried out at a B3LYP/6-311g-dp theory level clearly indicate that the co-existence of H_2_O molecules in the corrosion system causes a reduction in E_HOMO_ values for all investigated corrosion inhibitors. It means that the presence of H_2_O molecules in the corrosive environment reduces the anti-corrosion properties of investigated aliphatic Schiff bases as a result of the weakening of their ability to donate electrons to the surface of the protected metal. As a result of H_2_O molecules, the E_HOMO_ values of the aliphatic Schiff bases do not change significantly. Table S1 demonstrates that, for all compounds tested, the values of the change in HOMO orbital energy do not exceed 5% for any molecule tested. Among aliphatic Schiff bases with H atoms at the C atom (R_1_ and R_2_), an increase in the number of C atoms in the R_3_ substituent makes these molecules more sensitive to changes in the corrosive environment. Compared to the gas phase, the E_HOMO_ value of the N-MNI molecule was reduced by only 0.73%, while for the N-PMI molecule it was lowered by 2.67%. The presence of the -CH_3_ substituent at the N atom (R_3_) resulted in a greater decrease in E_HOMO_ values for the N-MEI and N-MB(1)I molecules, and a lower decrease in E_HOMO_ values for the N-MP(1)I molecule. This means that, for these corrosion inhibitors, there is no direct dependence of the weakening of the theoretical corrosion inhibition efficiency on the length of the aliphatic chain in the R_1_ substituent. However, such a correlation was observed for Schiff bases with the -C_2_H_5_ substituent at the N atom (R_3_). In these cases, the elongation of the R_1_ chain causes a higher weakening of the anticorrosive properties of Schiff bases, so they become more sensitive to the corrosive environment. For investigated corrosion inhibitors with a -C_3_H_7_ substituent at the N atom, an opposite relationship was observed. Table S1 indicates that Schiff bases with substituents -C_2_H_5_ and -C_3_H_7_ at the C atom (R_1_) are the least sensitive to a change in the corrosive environment. On the other hand, the highest weakening of anticorrosive properties occurring due to a change in the corrosive environment was observed for N-PEI, with the substituent -CH_3_ at the C atom (R_1_) amounting to almost 3%.

Definitely, the ability to accept electrons from protected metal surfaces of the investigated aliphatic Schiff bases is more sensitive to changes in the corrosive environment. Table S2 shows that, depending on the chemical structure of the inhibitor molecule, the aqueous environment leads to both enhancement and weakening of its electron-accepting properties. For Schiff bases with two H atoms attached to a C atom (R_1_ and R_2_) in an aqueous environment, a reduction in the LUMO orbital energy was observed. This means that compared to the gas phase, N-MMI, N-EMI and N-PMI molecules in aqueous phase will inhibit the corrosion process more effectively. Table S1, indicates also that with increasing the number of C atoms in the R_3_ substituent, the E_LUMO_ value of the investigated Schiff bases decrease by about 6% for N-MMI, about 27% for N-EMI and about 32% for N-PMI. These results indicate that an inhibitor with an R_3_ substituent containing more C atoms is characterized by better anti-corrosion properties. A similar but stronger relationship was observed for inhibitors with the -C_2_H_5_ substituent attached to the N atom (R_3_). In this case, the reduction in E_LUMO_ of the molecules (1Z)N-EEI, (1Z)N-EP(1)I and (1Z)N-EB(1)I due to a change in the corrosive environment from gaseous to aqueous was about 13%, 24% and 350%, respectively. However, there were no similar correlations noted for Schiff bases with the -CH_3_ substituent at the N atom (R_3_). In this case, a more than 2-fold increase in corrosion inhibition efficiency in aqueous media compared to the gas phase was observed, but only for the N-MP(1)I molecule. For the other two inhibitors (N-MEI and N-MB(1)I), an almost 2-fold and 10-fold reduction in the ability to accept electrons was observed, respectively. The combined effect of the aqueous environment on the corrosion inhibition efficiency of the studied Schiff bases was also observed for compounds with the -C_3_H_7_ substituent at the N atom (R_3_). In this case, presence of H_2_O molecule in corrosive medium caused a reduction in the electron-accepting properties of the N-PEI molecule and their enhancement for N-PP(1)I and N-PB(1)I molecules. This means that, compared to the gas phase, in an aqueous environment, inhibitor molecules which have a longer hydrocarbon chain attached to the C (R1) and N (R_3_) atoms are theoretically characterized by a greater ability to inhibit the corrosion process.

Compared to the studies conducted by Shenoy et al. [42], the corrosion inhibitors studied in this work have a higher EHOMO value and a significantly lower ELUMO value. This means that, compared to the aliphatic Schiff bases tested, they have a greater ability to accept and donate electrons, which is due to the presence of aromatic regions in their structure. Also, compared to the results obtained by Boukazoul et al. [43], the EHOMO values of aliphatic Schiff bases obtained in the aqueous phase are lower, and the ELUMO values of aliphatic Schiff bases are higher, indicating their lower corrosion inhibition efficiency.

**Figure 5:**
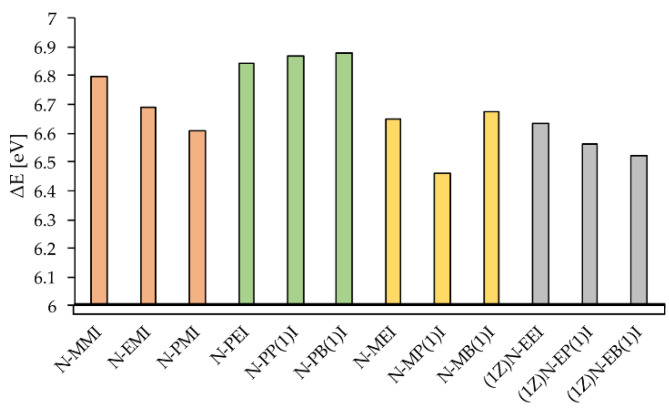
ΔE values of investigated aliphatic Schiff bases in aqueous phase.

Then, the effect of the aqueous corrosion environment on the chemical reactivity of the investigated corrosion inhibitors was analyzed. A comparison of the ΔE values determined for the tested Schiff bases in the gas phase (Figure 3) and the aqueous phase (Figure 5) shows that the dependence of chemical reactivity on the chemical structure of the studied inhibitors does not change. However, as it is shown in Table S2, in the aqueous phase, most of the investigated corrosion inhibitors become more reactive and therefore can more efficiently protect the surface of metals from the destructive effects of the corrosion process. It is worth pointing out that the percentage changes in ΔE are negligible, amounting to a maximum of about 2.5%. The exceptions are the N-MEI and N-MP(1)I molecules, where a decrease in chemical reactivity was observed respectively of 0.57% and 0.63%. These low changes can be ignored and it can be concluded that these inhibitors in the gas and aqueous phases are equally chemically reactive. Compared to the work [42], the obtained ΔE values shown in Figure 5 are about two times lower, indicating that inhibitors with aromatic regions in their structure more efficiently inhibit the corrosion process.

#### 3.1.3. Environment of HCl

**Figure 6:**
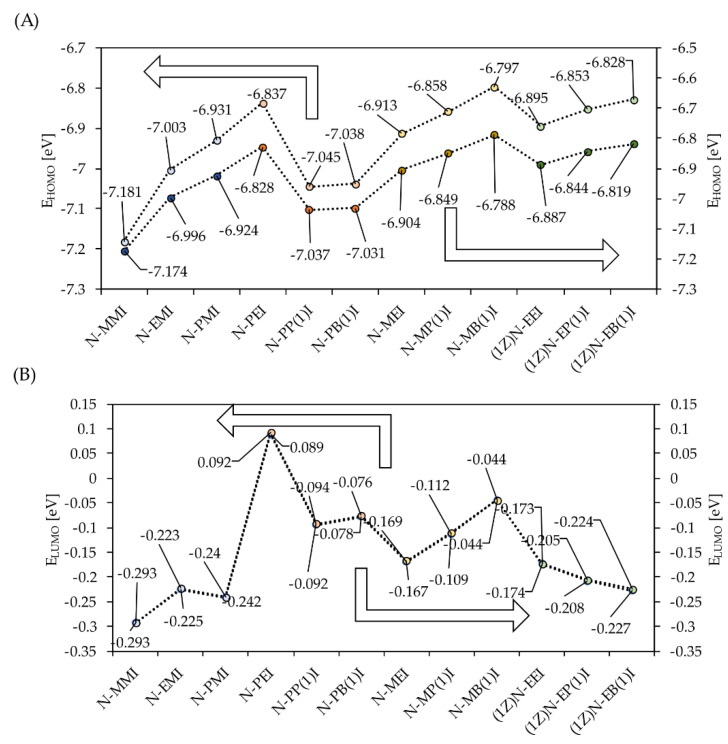
E_HOMO_ (**A**) and E_LUMO_ (**B**) of investigated Schiff bases in HCl solution with concentrations of 1 mol/dm^3^ (blue line) and 2 mol/dm^3^ (green line).

In the next step, it was studied how the presence of HCl molecules in the corrosive environment affects the anti-corrosive properties of aliphatic Schiff bases by changing their ability to accept and donate electrons. For this purpose, the E_HOMO_ and E_LUMO_ values of the studied corrosion inhibitors were determined in an HCl environments with concentrations of 1 mol/dm^3^ and 2 mol/dm^3^, and the obtained results are shown in Figure 6A,B. Comparison of these results to the gas phase indicates that, in both investigated HCl solutions, the dependence of the electron-donating and electron-accepting properties of the investigated inhibitors on their structure does not change.

Table S3 shows the changes in the E_HOMO_ values of the investigated aliphatic Schiff bases in an HCl environment with concentrations of 1 mol/dm^3^ and 2 mol/dm^3^, with respect to the values obtained in the gaseous and aqueous phases. The results clearly show that the acidic environment has the greatest effect in weakening the ability of the studied Schiff bases to donate electrons, without changing their dependence on the chemical structure. The maximum reduction in E_HOMO_ values relative to the gas phase was about 5% for both HCl solutions, with concentrations of 1 mol/dm^3^ and 2 mol/dm^3^. As is shown in Table S3, elongation of the hydrocarbon chain attached to the nitrogen atom (R_3_) for inhibitors, with two H atoms at the C atoms (R_1_ and R_2_), results in a proportionally higher reduction in the E_HOMO_ values of the investigated corrosion inhibitors. For N-MMI, the observed decrease was −1.11%, while for N-PMI the decrease was −3.20%. However, it is worth noting that the difference between the N-EMI and E-PMI molecules was insignificant at only 0.01%. A similar relationship was observed for corrosion inhibitors having a substituent-C_2_H_5_ at the C atom (R_1_). In contrast, an exactly opposite relationship was observed for compounds with the -C_3_H_7_ group at the C atom (R_1_). In this case, elongation of the hydrocarbon chain of R1 increases the resistance of the ability to donate electrons of the inhibitor molecule to changes in the corrosive environment. However, the presence of the -CH_3_ group at the N atom (R_3_) resulted in a greater decrease in E_HOMO_ values. For the N-MEI molecule, the decrease was 5.11%, for the N-MP(1)I molecule 4.37%, and for the N-MP(1)I molecule it was 4.49%. These values indicate that, the molecule with the -CH_3_ group attached to the C atom (R_1_) is the most sensitive to a change in the corrosive environment, and an increase in the number of C atoms in the R_1_ chain results in an increase in the resistance of the electron-donating properties of the investigated molecules to a change in the corrosive environment. Table S3 also shows that, for most of the studied aliphatic Schiff bases, the decrease in E_HOMO_ values did not exceed 1% compared to the aqueous phase. Only for the N-MP(1)I molecule was it 3.89%. Interestingly, a twofold increase in the HCl concentration of the corrosive medium results in a slight improvement in the electron-donating properties of the investigated Schiff bases, but this increase is negligible, with a maximum of 0.13%. Therefore, it can be neglected.

As shown in Figure 6B, the electron-accepting properties of the investigated corrosion inhibitors are comparable in an HCl solution with concentrations of 1 mol/dm^3^ and 2 mol/dm^3^. A comparison of the E_LUMO_ values with those shown in Figure 2 also indicates that the electron-accepting capacity from the surface of the protected metal, depending on the chemical structure of the inhibitor molecule, varies in exactly the same way as for the gas phase. However, there were some changes in the E_LUMO_ values relative to the gas phase, which are an indication of a strengthening or weakening of the electron-accepting properties in the HCl environment. As is shown in Table S4, for Schiff bases with H atoms at the C atom (N-MMI, N-EMI, N-PMI), with -C_2_H_5_ group at the N atom ((1Z)N-EEI, (1ZN-EP(1)I, (1Z)N-EN(1)I, and -C_3_H_7_ group at the N atom (N-PEI, N-PP(1)I, N-PB(1)I) there was an increase in their ability to accept electrons from the surface of the protected metal. It indicates their higher efficiency in inhibiting the corrosion process in the environment of HCl solution with concentrations of 1 mol/dm^3^ and 2 mol/dm^3^. For these groups of investigated corrosion inhibitors, a theoretical increase in the inhibition efficiency was observed with an increase in the number of C atoms in the aliphatic substituents R_3_ and R_1_. In contrast, a decrease in corrosion inhibition efficiency relative to the gas phase was observed only for the group of inhibitors tested containing the -CH_3_ substituent at the N atom (R3). In addition, quantum-chemical calculations indicated a weakening of the corrosion inhibition performance in comparison to the aqueous phase. As is shown in Table S4, an increase in E_LUMO_ values in comparison to the aqueous phase was observed for most of the studied compounds, what shows their lower corrosion inhibition efficiency.

DFT studies in an HCL medium were also carried out by Eze et al. [44] for 2-[(E)-(2,5-dimethoxybenzylidene) amino]-4-methylphenol (DMPC) bases. Two benzene rings and the substituent -CH_3_, -OH and -OCH_3_ are present in the structure of this compound. The EHOMO value obtained for this compound is higher, and the ELUMO value is lower, compared to the aliphatic Schiff bases studied in this work [44]. A comparison of the values shown in Figure 6 and in the paper indicates that, also, in the HCL environment, the presence of benzene rings positively affects the corrosion inhibition performance. In the work [44], inhibition of the co-corrosion process was obtained at the level of 87–97%, indicating that the values obtained for aliphatic Schiff bases should be lower.

**Figure 7:**
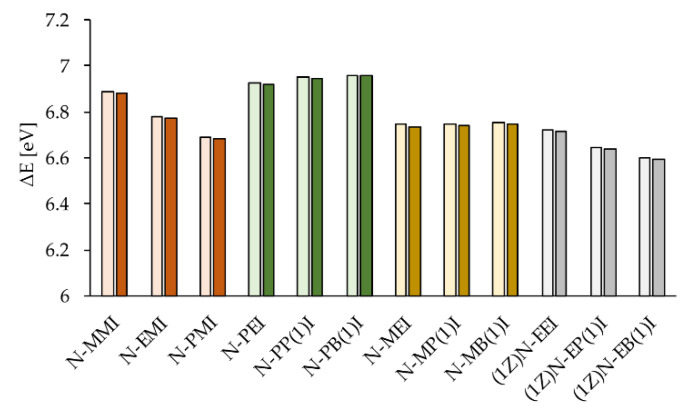
ΔE values of investigated aliphatic Schiff bases in environment of HCl with concentration 1 mol/dm^3^ (left bars) and 2 mol/dm^3^ (right bars).

Figure 7 shows ΔE values of investigated aliphatic Schiff bases in an HCl environment. Their comparison to values of ΔE determined in the gas phase (Figure 3) shows that, for most of the studied compounds, the dependence of the chemical reactivity on their structure is exactly the same. Table S5 shows that, compared to the gas and aqueous phases, the presence of HCl molecules in a corrosion system results in a lowering of the energy differences between the HOMO and LUMO orbitals of studied aliphatic Schiff bases. Therefore, it can be concluded that investigated corrosion inhibitors should protect the metal surface more effectively against corrosion in an acidic environment than in the gas and aqueous phases.

### 3.2. Dipole Moment (µ)

**Figure 8:**
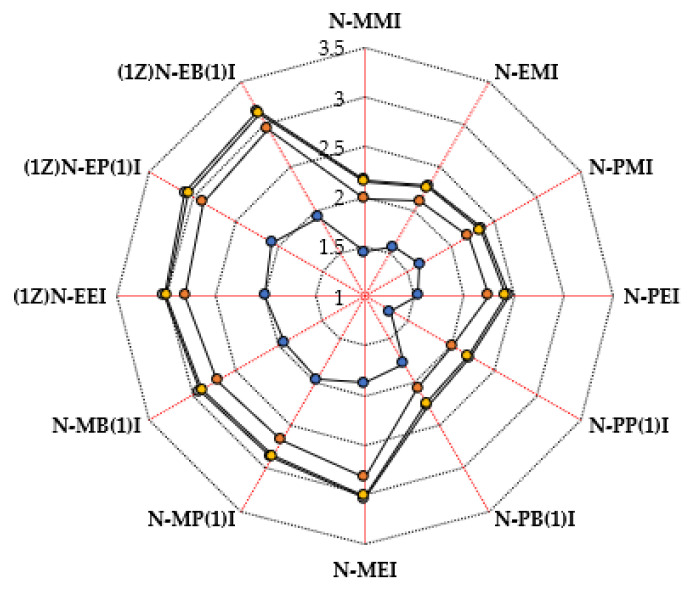
The dipole moment of the investigated Schiff bases in the gas phase (blue line), aqueous phase (orange line) and HCl environment with a concentration of 1 mol/dm^3^ (yellow line) and 2 mol/dm^3^ (grey line).

Recent results also indicate a dependence of the corrosion inhibition efficiency on the dipole moment of the inhibitor molecule. Both theoretical and experimental studies have shown that a higher value of dipole moment favors the accumulation of inhibitor molecules on the surface of the protected metal, while limiting the degree of corrosion. As shown in Figure 8, the investigated aliphatic Schiff bases with different chemical structures are characterized by different dipole moments. In all the analyzed corrosion environments, an increase in the dipole moment was observed with an increase in the number of C atoms forming the R_3_ hydrocarbon chain. This means that, among molecules with H atoms at the C atom (R_1_ and R_2_), the N-PMI molecule will be the most efficient in limiting the corrosion process. Exactly the same relationship was observed for N-MEI, N-MP(1)I and N-MB(1)I molecules with a -CH_3_ substituent at the N atom (R_3_) in the gas and aqueous phases. Interestingly, quantum chemical calculations carried out at B3LYP/6-311g-dp theory level indicated that this trend changes due to the presence of HCl in the corrosive environment. In both HCl environments with concentration of 1 mol/dm^3^ and 2 mol/dm^3^, an increase in the R_1_ chain length resulted in a decrease in the dipole moment, so that in the HCl environment, among compounds with a -CH_3_ group at the N atom, the N-MEI molecule will have the highest corrosion inhibition efficiency. Another trend was observed for compounds with a -C_2_H_5_ group at the N atom. In the gas phase, for this group of investigated compounds, a decrease in their dipole moment of about 4.5% was observed with an extension of the R_1_ chain from one to three C atoms. In contrast, in the other corrosion environments, this resulted in an increase in the dipole moment and therefore an increase in the corrosion inhibition efficiency. In all analyzed corrosion environments, the elongation of the R_1_ chain of inhibitors having a -C_3_H_7_ substituent at the N atom (R_3_) resulted in an increase in the corrosion inhibition efficiency due to an increase in their dipole moment.

### 3.3. Number of Transferred Electrons (ΔN)

**Figure 9:**
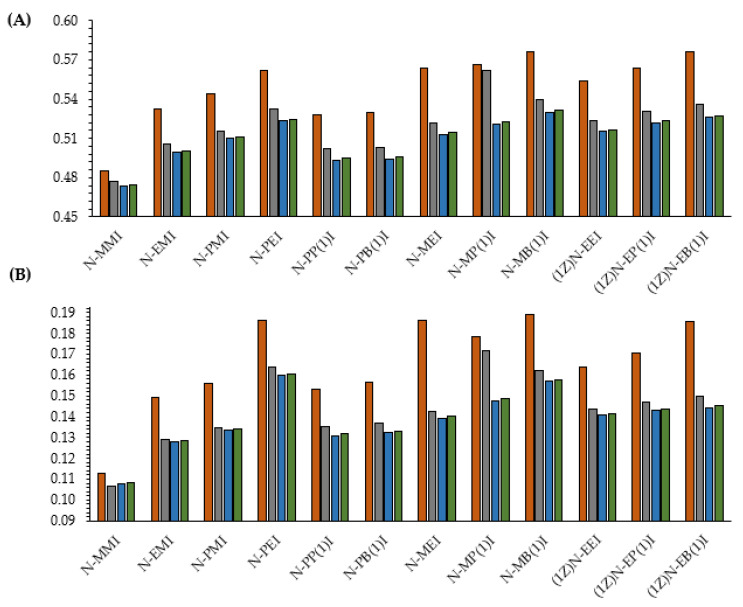
ΔN transferred during corrosion protection of Fe (**A**) and Cu (**B**) in gaseous (orange bars), aqueous (grey bars), HCl of 1 mol/dm^3^ (blue bars) and HCl of 2 mol/dm^3^ (green bars) environments.

The value of ΔN shows how electrons are transferred during corrosion protection of metals. In the case of ΔN > 0, the corrosion inhibitor is an electron donor; in the case of ΔN < 0, the corrosion inhibitor is an electron acceptor. The ΔN value is commonly used in evaluating the corrosion properties of organic compounds [24,45,46]. As indicated by the results of a study conducted by Lukovits et al. [47], if N < 3.6 then an increase in this value suggests an increase in anticorrosive properties. This analysis was carried out for all analyzed corrosion environments independently for two structural metals: iron (Fe) and copper (Cu). Figure 9A,B show that ΔN is positive for all the investigated corrosion inhibitors in all the evaluated corrosion environments. This means that during the anti-corrosion process in all investigated aliphatic Schiff bases electron is transfered from the corrosion inhibitor molecule to protected metal surface. A higher value of ΔN indicates stronger metal-inhibitor corrosion interactions and, consequently, a higher corrosion inhibition efficiency. Figure 9A,B clearly indicate that the electron transfer process occurs more efficiently with the Fe surface than with Cu surface. Quantum-chemical calculations carried out at the B3LYP/6-311g-dp-theory level indicated exactly the same dependence of ΔN on the corrosion inhibitor’s chemical structure with respect to both studied metals. ΔN increased for both Fe and Cu surfaces, as the number of carbon atoms in the R_3_ hydrocarbon chain increased, for molecules with two H atoms at the C atom (R_1_ and R_2_). This increase is significantly higher during Cu corrosion protection and is about 38% (for Fe protection it is about 12%). The hydrocarbon chain length of R_1_ has a much smaller effect on the ΔN value when the substituent R_3_ is formed by the -CH_3_, -C_2_H_5_ and -C_3_H_7_ groups. For the corrosion inhibitors with substituents -CH_3_, -C_2_H_5_, increases in ΔN values of 2.7% and 4.10% were observed for Fe, and 1.4% and 13.4% for Cu, respectively. In contrast, for both analyzed metals, decreases in ΔN values were observed during R_1_ hydrocarbon chain elongation for molecules with a -C_3_H_7_ group at the N atom (R_3_). This decrease was 5.6% and 1.6% for Fe and Cu, respectively.

Furthermore, Figure 9A,B and Table S6 clearly show the significant influence of the corrosion environment on the ΔN value. Both aqueous and HCl environments reduce the electron transfer efficiency at the metal–corrosion medium interface. This is probably due to competitive adsorption of H_2_O and HCl molecules on the surface of the protected metal. As DFT/B3LYP/6-311g-dp calculations showed, a smaller decrease was observed during Fe protection. The percentage decrease in ΔN in the aqueous medium relative to the gas phase ranged from 0.75% (for the N-MB(1)I molecule) to 7.03% (for the (1Z)N-EB(1)I molecule). A higher reduction in the inhibition efficiency of the corrosion process relative to the gas phase was observed in an HCl environment with concentrations of 1 mol/dm^3^ and 2 mol/dm^3^. In the first case, the decrease in ΔN ranged from 2.34% (for the (N-MMI) molecule) to 8.94% (for the (N-MEI) molecule), while in the second case it ranged from 2.11% (for the N-MMI molecule) to 8.70% (for the N-MEI molecule). The reduction in ΔN values was significantly lower relative to the aqueous phase in both the 1 mol/dm^3^ HCl medium (ranged from 0.76% for the N-MMI molecule to 7.25% for the N-MP(1)I molecule) and the 2 mol/dm^3^ HCl environment (ranged from 0.55% for the N-MMI molecule to 7.01% for the N-MP(1)I molecule). A twofold increase in HCl concentration resulted in a slight increase in the value of only about 0.20%, clearly demonstrating that the number of HCl molecules in the corrosive environment does not affect the electron transfer between the inhibitor molecule and the surface of the protected metal. A greater reduction in ΔN with a change in corrosion environment was observed during corrosion protection of the Cu surface. Compared to the gas phase, a decrease in ΔN ranging from 3.75% (for the N-MP(1)I molecule) to 19.54% (for the (1Z)N-EB(1)I molecule) was observed in the aqueous medium. Even higher decreases were observed in the HCl environment. Its presence in the corrosion system in an amount of 1 mol/dm^3^ resulted in a decrease in ΔN of up to 25.29% for the N-MEI molecule. Comparable decreases were observed for HCl with concentration 2 mol/dm^3^.

### 3.4. Initial Metal–Molecule Interaction Energy (Δψ)

**Figure 10:**
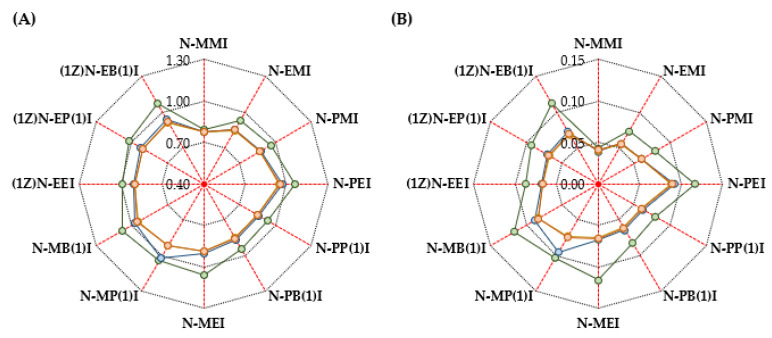
Initial Fe (**A**)/Cu (**B**)—studied Schiff bases interaction energy (Δψ).

Another very important parameter in the evaluation of anti-corrosion properties is the initial metal–molecule interaction energy (Δψ). It describes the strength of the interactions of the tested corrosion inhibitor with the surface of the protected metal. So, a higher value of |Δψ| will indicate definitely stronger inhibitor–metal surface interactions, and consequently theoretically higher corrosion inhibition efficiency. Figure 10A,B clearly indicate stronger interactions of the investigated Schiff bases with the Fe surface than with the Cu surface. As indicated by quantum chemical calculations carried out at B3LYP/6-311g-dp theory level, an increase in the length of the hydrocarbon chain attached to the N atom (R_3_) in the presence of hydrogen atoms at the C atom (R_1_ and R_2_) promotes improved interactions of aliphatic Schiff bases with the Fe surface. This is indicated by an approximately 20% increase in |Δψ|. Despite the significantly weaker interactions, this effect is more noticeable in the case of the interactions of the investigated aliphatic Schiff bases with the Cu surface, where an increase in the |Δψ| value of about 80% was noted. As is shown in Figure 10A,B, the same dependence was observed during the elongation of the hydrocarbon chain R_1_ in the presence of the -CH_3_ and -C_2_H_5_ substituent at the N atom (R_3_). For Schiff bases with the -CH_3_ group, an increase in the interaction strength with the Fe surface of 1.84% and with the Cu surface of 0.15% was observed. For investigated corrosion inhibitors with the -C_2_H_5_ substituent, an enhancement of the interaction strength with the Fe and Cu surfaces of more than 8% and 28%, respectively, was observed. A completely opposite relationship, namely a decrease in the strength of the interactions of the studied corrosion inhibitors with an increase in the number of C atoms forming the substituent R_1_, was observed for compounds with the substituent -C_3_H_7_ at the N atom (R_3_). This decrease with respect to Fe and Cu was about 10% and 30%, respectively. Figure 10A,B and Table S7 indicate a weakening of the interaction energies of all investigated aliphatic Schiff bases both as a result of changing the corrosion environment to H_2_O and HCl. Compared to the gas phase, the aqueous environment causes a different degree of decrease in |Δψ| depending on the chemical structure of the corrosion inhibitor. In case of Fe surface, this decrease varied from about 2% (for the N-MP(1)I molecule) to almost 15% (for the N-MEI molecule). More siginificant energy losses in the aqueous phase ranging from almost 8% (for the N-MP(1)I molecule) to more than 41% (for the N-MEI molecule) were observed for the interactions of the investigated corrosion inhibitors with the Cu surface. Moreover, the introduction in corrosion environement of HCl solution with concentration of 1 mol/dm^3^ resulted in a further weakening of the interactions of the studied Schiff bases with both Fe and Cu surfaces. The observed weakening of the interaction energies with the Fe surface ranged from almost 3% for the N-MMI molecule to more than 16% for the N-MEI molecule. While, for Cu more significant reductions, ranging from almost 7% to over 38%, were observed. Compared to the aqueous phase, the introduction of HCl molecules resulted in smaller changes in the interaction energies of the studied corrosion inhibitors with the surface of the protected metals (Fe and Cu). As indicated in Figure 10A,B and Table S7, a two-fold increase in HCl concentration in the corrosion system does not result in a further significant decrease in the interaction energies of the analyzed corrosion inhibitors with the Fe and Cu surfaces.

### 3.5. Electrostatic Interactions of Investigated Aliphatic Schiff Bases

A barrier layer of corrosion inhibitor molecules can also be formed by physical adsorption. Electrostatic interactions of negatively charged N atoms of inhibitor molecules with positively charged atoms of the protected metal can be used to investigate the ‘strength’ of physical adsorption and, consequently, the effectiveness of corrosion inhibition. Table 2 shows the values of the N atoms partial charges of the investigated corrosion inhibitors molecules determined by Mulliken population analysis. The obtained results indicate that the elongation of the hydrocarbon chain at the N atom (R_3_) caused a decrease in its partial charge in all analyzed corrosion environments. This means that molecules with a longer R_3_ chain will more strongly electrostatically interact with the positively charged surface of the protected metal, i.e., will inhibit the corrosion process more efficiently. The partial charges of the N atom of N-MMI, N-EMI and N-PMI molecules indicate that the strength of their electrostatic interactions with the surface of the protected metal increases in the water phase and HCl environment by 22% and 45%, respectively. Mulliken population analysis of N-MEI, N-MP(1)I and N-MB(1)I molecules indicated that the presence of the -CH_3_ substituent at the N atom (R_3_) causes a decrease in its partial charge and a simultaneous increase in electrostatic interactions. However, for this group of investigated inhibitors, no dependence of the partial charge of N on the length of the hydrocarbon chain forming the R1 substituent was observed. Stronger electrostatic interactions in all corrosion media tested were also observed for inhibitors with the -C_2_H_5_ substituent at the N atom (R_3_). In contrast to inhibitors with the -CH_3_ substituent, for this group of tested compounds it was observed that the N atoms of the (1Z)N-EP(1)I and (1Z)N-EB(1)I molecules have the most negative charge, so they can interact most strongly with the positively charged surface of the protected metal. In addition, comparable charges were determined for Schiff bases with the -C_3_H_7_ substituent at the N atom.

## 4. Conclusions

DFT methods are an excellent modern tool used in the design of new corrosion inhibitors. Their use allows researchers to estimate the efficiency of inhibition of the corrosion process by organic compounds on the basis of their electronic structure with satisfactory accuracy. In addition, the use of quantum-chemical methods makes it possible to study the interactions of the inhibitor molecule with the surface of the protected metal. The results of quantum-chemical calculations presented in this work lead to the following conclusions:

1. Among the investigated aliphatic Schiff bases, the N-MP(1)I molecule with a high HOMO orbital energy, a relatively low LUMO orbital energy, a low energy difference between them and a high dipole moment value has the theoretically highest corrosion inhibition efficiency.
2. This indicates that the molecule has a high ability to accept and donate electrons during the formation of a protective layer. This is very important because the DFT calculations indicate that this molecule can protect the metal surface by forming electron transfer. This is also confirmed by the ΔN value determined for both Fe and Cu. Its high value is likely due to the structure of N-MP(1)I. In this molecule the N atom, which is the main source of electrons exchanged with the surface of the protected metal, is the most exposed, which greatly facilitates this process. This can also be linked to its high reactivity. On the other hand, the high polarity of the molecule is most likely due to the presence of the substituent -C_2_H_5_ whose H atoms with positive partial charge cause a high gradient in charge distribution, resulting in high polarity. Compared to the gas phase, the anticorrosion properties of all investigated inhibitors in the aqueous phase and in the HCl environment are lower, including their ability to donate and accept electrons. This is probably due to the competitive interaction of H_2_O and HCl molecules with the surface of the protected metal.
3. The concentration of the HCl solution in the corrosive environment does not significantly affect the ability of the molecules of the tested corrosion inhibitors to accept and donate electrons, their polarity, the number of transferred electrons and the energy of the interaction between the inhibitor and the protected metal.
4. During the corrosion protection of investigated aliphatic Schiff bases, electron transfer occurs from the inhibitor molecule to the surface of the protected metal in all studied corrosion environments.
5. During the formation of a protective layer by the studied inhibitors, a greater number of electrons are transferred to the Fe surface; moreover, electron transfer to the Cu surface is more sensitive to changes in the corrosion environment.
6. In the gas phase, the protective corrosion layer is formed mainly by chemisorption, while in the aqueous environment and HCl solution, the contribution of physical adsorption increases, as indicated by stronger electrostatic interactions.

## Figures and Tables

**Table 1:** Chemical names, structures and abbreviations of the new corrosion inhibitors investigated in the paper.

Name	Chemical Structure	Abbreviation
N-methylmethanimine	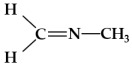	N-MMI
N-ethylmethanimine	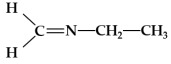	N-EMI
N-propylmethylimine	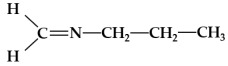	N-PMI
N-propylethylimine	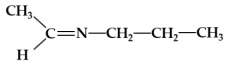	N-PEI
N-propylpropan-1-imine	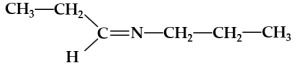	N-PP(1)I
N-propylbutan-1-imine	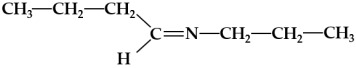	N-PB(1)I
N-methyletanimine	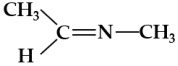	N-MEI
N-methylpropan-1-imine	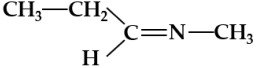	N-MP(1)I
N-methylbutan-1-imine	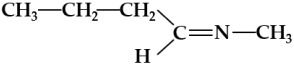	N-MB(1)I
(1Z)-N-ethyletanimine	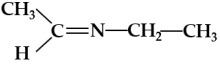	(1Z)N-EEI
(1Z)-N-ethylpropan-1-imine	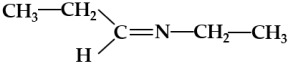	(1Z)N-EP(1)I
(1Z)-ethylbutan-1-imine	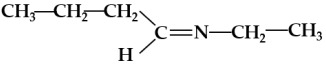	(1Z)N-EB(1)I

**Table 2:** Partial charges of the N atoms of the aliphatic Schiff bases under study determined by Mulliken population analysis.

	Gaz Phase	H_2_O	1M HCl	2M HCl
N-MMI	−0.244	−0.314	−0.353	−0.350
N-EMI	−0.254	−0.324	−0.364	−0.362
N-PMI	−0.257	−0.327	−0.369	−0.365
N-PEI	−0.278	−0.348	−0.392	−0.389
N-PP(1)I	−0.266	−0.330	−0.369	−0.367
N-PB(1)I	−0.266	−0.329	−0.370	−0.366
N-MEI	−0.272	−0.343	−0.389	−0.385
N-MP(1)I	−0.273	−0.347	−0.391	−0.388
N-MB(1)I	−0.273	−0.346	−0.389	−0.386
(1Z)N-EEI	−0.266	−0.339	−0.384	−0.380
(1Z)N-EP(1)I	−0.275	−0.347	−0.390	−0.387
(1Z)N-EB(1)I	−0272	−0.347	−0.389	−0.390

## Data Availability

Not applicable.

## Supplementary Materials

The following supporting information can be downloaded at: https://www.mdpi.com/article/10.3390/ma15196725/s1, Table S1. Percentage change of E_HOMO_ and E_LUMO_ values in the aqueous phase. Table S2. Percentage change of ΔE values in the aqueous phase. Table S3. Percentage change of E_HOMO_ values in the HCl environment. Table S4. Percentage change of E_LUMO_ values in the HCl environment. Table S5. Percentage change of ΔE values in the HCl environment. Table S6. Percentage change of ΔN values in three different corrosion environments. Table S7. Percentage change of ΔΨ values in three different corrosion environments.
