# Supplementary material for: Computational Design of Anticorrosion Properties of Novel, Low-Molecular Weight Schiff Bases
Source: Materials (Basel). 2022 Sep 27;15(19):6725. doi: 10.3390/ma15196725 (PMC9573614; doi:10.3390/ma15196725)
Supplement: Supplementary file 1 [file materials-15-06725-s001.zip › materials-1911432-supplementary.pdf]

Supplementary Materials for

Computational design of anticorrosion properties of novel, low-molecular weight Schiff Bases.

Szymon Malinowski [1*]

[1]Lublin University of Technology, Faculty of Civil Engineering and Architecture, Department of Construction Materials Engineering and Geoengineering, Nadbystrzycka 40, 20-618 Lublin, Poland,

*Correspondence: s.malinowski@pollub.pl; Tel.: 81 538 4451

Table S1. Percentage change of E_{HOMO} and E_{LUMO} values in the aqueous phase.

	Change [%]	
	E_{HOMO}	E_{LUMO}
N-MMI	-0,73	-6,61
N-EMI	-2,61	-27,36
N-PMI	-2,67	-32,11
N-PEI	-2,93	49,60
N-PP(1)I	-2,55	-40,48
N-PB(1)I	-2,65	-102,04
N-MEI	-4,23	289,91
N-MP(1)I	-0,46	-102,90
N-MB(1)I	-3,54	1100,00
(1Z)N-EEI	-2,89	-13,30
(1Z)N-EP(1)I	-3,13	-24,34
(1Z)N-EB(1)I	-3,98	-356,60

Table S2. Percentage change of ΔE values in the aqueous phase.

	Change [%]
N-MMI	-0,44
N-EMI	-1,81
N-PMI	-1,68
N-PEI	-1,95
N-PP(1)I	-2,08
N-PB(1)I	-1,93
N-MEI	0,57
N-MP(1)I	0,63
N-MB(1)I	-2,52
(1Z)N-EEI	-2,58
(1Z)N-EP(1)I	-2,50
(1Z)N-EB(1)I	-1,08

Table S3. Percentage change of E_{HOMO} values in the HCl environment.

	Change [%]				
	1M HCl		2M HCl		
	Comparison to gas phase	Comparison to water phase	Comparison to gas phase	Comparison to water phase	Comparison to HCl solution with concentration of 1 mol/dm^3
N-MMI	-1.11	-0.38	-1.01	-0.28	0.10
N-EMI	-3.21	-0.59	-3.11	-0.49	0.10
N-PMI	-3.20	-0.52	-3.10	-0.42	0.10
N-PEI	-3.80	-0.84	-3.66	-0.71	0.13
N-PP(1)I	-3.41	-0.83	-3.29	-0.72	0.11
N-PB(1)I	-3.50	-0.83	-3.40	-0.73	0.10
N-MEI	-5.11	-0.85	-4.97	-0.71	0.13
N-MP(1)I	-4.37	-3.89	-4.23	-3.76	0.13
N-MB(1)I	-4.49	-0.92	-4.35	-0.79	0.13
(1Z)N-EEI	-3.64	-0.73	-3.52	-0.61	0.12
(1Z)N-EP(1)I	-3.99	-0.84	-3.85	-0.71	0.13
(1Z)N-EB(1)I	-4.93	-0.92	-4.79	-0.78	0.13

Table S4. Percentage change of E_{LUMO} values in the HCl environment.

	Change [%]				
	1M HCl		2M HCl		
	Comparison to gas phase	Comparison to water phase	Comparison to gas phase	Comparison to water phase	Comparison to HCl solution with concentration of 1 mol/dm^3
N-MMI	12.01	17.46	12.01	17.46	0.00
N-EMI	-6.13	16.67	-5.19	17.41	0.89
N-PMI	-11.01	15.97	-10.09	16.67	0.83
N-PEI	28.80	-41.27	26.40	-46.03	-3.37
N-PP(1)I	-11.90	20.34	-9.52	22.03	2.13
N-PB(1)I	-59.18	21.21	-55.10	23.23	2.56
N-MEI	255.05	18.36	253.21	19.32	1.18
N-MP(1)I	-62.32	20.00	-57.97	22.14	2.68
N-MB(1)I	833.33	26.67	833.33	26.67	0.00
(1Z)N-EEI	7.45	18.31	7.98	18.78	0.57
(1Z)N-EP(1)I	-10.05	11.49	-8.47	12.77	1.44
(1Z)N-EB(1)I	-328.30	6.20	-322.64	7.44	1.32

Table S5. Percentage change of ΔE values in the HCl environment.

	Change [%]				
	1M HCl		2M HCl		
	Comparison to gas phase	Comparison to water phase	Comparison to gas phase	Comparison to water phase	Comparison to HCl solution with concentration of 1 mol/dm^3
N-MMI	-1.76	-1.31	-1.65	-1.31	-0.10
N-EMI	-3.12	-1.29	-3.04	-1.29	-0.07
N-PMI	-2.94	-1.24	-2.86	-1.24	-0.07
N-PEI	-3.19	-1.21	-3.10	-1.21	-0.09
N-PP(1)I	-3.30	-1.19	-3.21	-1.19	-0.09
N-PB(1)I	-3.10	-1.15	-3.02	-1.15	-0.07
N-MEI	-0.87	-1.44	-0.76	-1.44	-0.10
N-MP(1)I	-3.75	-4.41	-3.66	-4.41	-0.09
N-MB(1)I	-3.72	-1.17	-3.58	-1.17	-0.13
(1Z)N-EEI	-3.96	-1.34	-3.85	-1.34	-0.10
(1Z)N-EP(1)I	-3.81	-1.28	-3.72	-1.28	-0.09
(1Z)N-EB(1)I	-2.28	-1.18	-2.18	-1.18	-0.09

Table S6. Percentage change of ΔN values in three different corrosion environments.

Protected surface →	Fe					
Corrosion medium →	water	1M HCl		2M HCl		
	Comparison to gas phase	Comparison to water phase	Comparison to gas phase	Comparison to water phase	Comparison to HCl solution with concentration of 1 mol/dm^3	Comparison to HCl solution with concentration of 1 mol/dm^3
N-MMI	-1.56	-2.31	cz	-2.11	-0.55	0.21
N-EMI	-5.07	-6.22	-1.21	-6.03	-1.01	0.21
N-PMI	-5.12	-6.14	-1.08	-5.95	-0.88	0.21
N-PEI	-5.23	-6.77	-1.62	-6.53	-1.37	0.25
N-PP(1)I	-4.91	-6.49	-1.67	-6.27	-1.44	0.23
N-PB(1)I	-5.04	-6.62	-1.66	-6.44	-1.46	0.20
N-MEI	-7.36	-8.94	-1.71	-8.70	-1.45	0.26
N-MP(1)I	-0.75	-7.94	-7.25	-7.70	-7.01	0.26
N-MB(1)I	-6.31	-7.98	-1.79	-7.74	-1.53	0.26
(1Z)N-EEI	-5.47	-6.87	-1.48	-6.65	-1.25	0.23

(1Z)N-EP(1)I	-5.84	-7.43	-1.69	-7.19	-1.43	0.26
(1Z)N-EB(1)I	-7.03	-8.73	-1.83	-8.49	-1.57	0.26
Protected Surface →	Cu					
Corrosion medium →	water	1M HCl		2M HCl		
	Comparison to gas phase	Comparison to water phase	Comparison to gas phase	Comparison to water phase	Comparison to HCl solution with concentration of 1 mol/dm^3	Comparison to HCl solution with concentration of 1 mol/dm^3
N-MMI	-5.27	-4.24	1.09	-3.69	-1.67	0.57
N-EMI	-13.54	-14.44	-1.04	-13.93	-0.45	0.59
N-PMI	-13.74	-14.32	-0.67	-13.82	-0.10	0.58
N-PEI	-11.93	-14.19	-2.56	-13.64	-1.95	0.63
N-PP(1)I	-11.92	-14.55	-2.99	-14.01	-2.37	0.64
N-PB(1)I	-12.58	-15.27	-3.08	-14.80	-2.54	0.56
N-MEI	-23.40	-25.29	-2.46	-24.77	-1.79	0.69
N-MP(1)I	-3.75	-17.33	-14.11	-16.75	-13.51	0.69
N-MB(1)I	-14.19	-16.98	-3.26	-16.52	-2.71	0.56
(1Z)N-EEI	-12.50	-14.16	-1.89	-13.66	-1.33	0.58
(1Z)N-EP(1)I	-13.71	-16.13	-2.80	-15.52	-2.10	0.72
(1Z)N-EB(1)I	-19.54	-22.39	-3.55	-21.83	-2.85	0.72

Table S7. Percentage change of ΔΨ values in three different corrosion environments.

Protected metal →	Fe					
Corrosion medium →	water	1M HCl		2M HCl		
	Comparison to gas phase	Comparison to water phase	Comparison to gas phase	Comparison to water phase	Comparison to HCl solution with concentration of 1 mol/dm^3	Comparison to HCl solution with concentration of 1 mol/dm^3
N-MMI	-2.67	-2.89	-0.22	-2.58	0.09	0.32
N-EMI	-8.26	-9.32	-1.15	-9.01	-0.82	0.34
N-PMI	-8.46	-9.32	-0.94	-9.01	-0.60	0.34
N-PEI	-8.44	-10.30	-2.04	-9.93	-1.63	0.42
N-PP(1)I	-7.69	-9.68	-2.15	-9.34	-1.78	0.38
N-PB(1)I	-8.10	-10.11	-2.19	-9.81	-1.86	0.33
N-MEI	-14.67	-16.36	-1.99	-16.01	-1.58	0.42

N-MP(1)I	-2.11	-12.07	-10.17	-11.69	-9.79	0.43
N-MB(1)I	-10.00	-12.18	-2.41	-11.84	-2.04	0.39
(1Z)N-EEI	-8.34	-9.84	-1.64	-9.51	-1.28	0.36
(1Z)N-EP(1)I	-9.13	-11.05	-2.11	-10.66	-1.68	0.44
(1Z)N-EB(1)I	-12.63	-14.80	-2.49	-14.43	-2.06	0.44
Protected metal ⟶	Cu					
Corrosion medium ⟶	water	1M HCl		2M HCl		
	Comparison to gas phase	Comparison to water phase	Comparison to gas phase	Comparison to water phase	Comparison to HCl solution with concentration of 1 mol/dm^3	Comparison to HCl solution with concentration of 1 mol/dm^3
N-MMI	-9.87	-6.69	3.53	-5.71	4.61	1.05
N-EMI	-23.89	-24.51	-0.81	-23.66	0.30	1.12
N-PMI	-24.34	-24.42	-0.11	-23.60	0.97	1.09
N-PEI	-20.92	-24.01	-3.91	-23.12	-2.78	1.18
N-PP(1)I	-20.80	-24.57	-4.77	-23.68	-3.64	1.19
N-PB(1)I	-22.10	-25.99	-4.99	-25.21	-3.99	1.05
N-MEI	-41.66	-43.70	-3.49	-42.97	-2.25	1.28
N-MP(1)I	-7.94	-29.09	-22.97	-28.16	-21.97	1.30
N-MB(1)I	-24.51	-28.52	-5.31	-27.81	-4.38	0.99
(1Z)N-EEI	-21.46	-23.40	-2.46	-22.58	-1.43	1.06
(1Z)N-EP(1)I	-23.68	-26.97	-4.31	-25.98	-3.01	1.36
(1Z)N-EB(1)I	-34.56	-38.40	-5.87	-37.56	-4.59	1.36
